# Ultrafast 27 GHz cutoff frequency in vertical WSe_2_ Schottky diodes with extremely low contact resistance

**DOI:** 10.1038/s41467-020-15419-1

**Published:** 2020-03-27

**Authors:** Sung Jin Yang, Kyu-Tae Park, Jaeho Im, Sungjae Hong, Yangjin Lee, Byung-Wook Min, Kwanpyo Kim, Seongil Im

**Affiliations:** 1 Department of Physics, Van der Waals Materials Research Center, Yonsei University, Seoul, 03722 Republic of Korea; 2 Department of Electrical and Electronic Engineering, Yonsei University, Seoul, 03722 Republic of Korea; 3 Department of Electrical Engineering and Computer Science, The University of Michigan, Ann Arbor, MI 48109 USA

**Keywords:** Electrical and electronic engineering, Electronic devices

## Abstract

Ultra-thin two-dimensional semiconducting crystals in their monolayer and few-layer forms show promising aspects in nanoelectronic applications. However, the ultra-thin nature of two-dimensional crystals inevitably results in high contact resistance from limited channel/contact volume as well as device-to-device variability, which seriously limit reliable applications using two-dimensional semiconductors. Here, we incorporate rather thick two-dimensional layered semiconducting crystals for reliable vertical diodes showing excellent Ohmic and Schottky contacts. Using the vertical transport of WSe_2_, we demonstrate devices which are functional at various frequency ranges from megahertz AM demodulation of audio signals, to gigahertz rectification for fifth-generation wireless electronics, to ultraviolet–visible photodetection. The WSe_2_ exhibits an excellent Ohmic contact to bottom platinum electrode with record-low contact resistance (~50 Ω) and an exemplary Schottky junction to top transparent conducting oxide electrode. Our semitransparent vertical WSe_2_ Schottky diodes could be a key component of future high frequency electronics in the era of fifth-generation wireless communication.

## Abstract (web summary)

Two-dimensional materials show promise for 5G wireless communication applications. Here, the authors report vertical Schottky diodes based on thick mechanically exfoliated WSe_2_ flakes having low ohmic contact resistance of 50 Ω and ultrafast cutoff frequency of 27 GHz.

## Introduction

Layered semiconducting crystals have been extensively studied in many aspects of scientific features and device applications^1–3^. Representative transition metal dichalcogenides (TMDs) and black phosphorus have been used for device applications, such as field effect transistors (FETs)^4–7^ and PN diodes^8,9^, complementary metal oxide semiconductor inverters^10^, photovoltaic diode circuits^11,12^, FET circuits for light emitting diodes^13,14^, and ring oscillators. Most of the previous devices have utilized the lateral electrical transport as well as the ultra-thin nature of semiconducting two-dimensional (2D) crystals. However, the ultra-thin nature of 2D crystals inevitably results in high contact resistance from limited channel/contact volume. Moreover, the issues from surface adsorbates and defects introduced during device fabrication processing become more pronounced as we deal with thinner 2D crystals. Because of these technical challenges, the reliable applications using ultra-thin 2D semiconductors, especially in ultrahigh frequencies, have been extremely difficult, although a few recent attempts show ~10 GHz of state-of-the-art operating frequency in 2D-like thin TMD Schottky diode^15,16^.

The vertical transistors and diodes could be alternative device designs for 2D electronic applications. Vertically stacked layered crystals have been used in different device designs and have shown great advantages over lateral devices, such as tunneling or ultrafast charge transfer-mediated device operation^8,17–22^. The band alignment through a combination of 2D crystals and contact engineering in these vertical devices is very crucial to achieve high-performance electron devices^23–25^. In particular, contact engineering to van der Waals 2D semiconductors is timely important, whether the device structure is for the lateral or vertical direction. Even though recent works using van der Waals contacts of metals or graphene electrodes to 2D semiconductors provided potentially useful methods to engineer contact properties^26–30^, the realization of the ultra-low contact resistance together with ultrahigh frequency in vertical device geometry has yet to be reported.

In the present study for high frequency devices, we circumvent the serious parasitic resistance issues of 2D semiconductors by incorporating rather thick 2D layered TMD and achieving excellent Ohmic and Schottky contacts in vertical diodes. We find that, in a vertical diode, relatively thick (a few tens of nanometer) TMD can serve as an ideal platform for achieving ultra-low contact resistance and capacitance. We demonstrate that excellent Ohmic contact using semitransparent platinum (Pt) to WSe_2_ with record-low series resistance (~50 Ω) and low specific contact resistance of 3.4 × 10^−5^ Ω cm^2^. The top transparent electrode (indium tin oxide: ITO) on WSe_2_ serves as an exemplary Schottky junction. Some other part of the top surface is electrically isolated by thin aluminum oxide (Al_2_O_3_), which becomes a self-aligned parallel capacitor suitable for amplitude modulation (AM) demodulation circuits on glass substrate. More importantly, by modulating the thick 2D layered crystals in the vertical diodes with capacitive dimension, very low series resistance and capacitance (0.145 pF) are simultaneously achieved, allowing an ultrahigh cutoff frequency up to record high 27.1 GHz^22,31^. Equipped with practically facile fabrication process using thick and large-area van der Waals TMD crystals, our vertical TMD Schottky diodes exhibit high device stability and reproducibility. In fact, such vertical Schottky or PN diodes have been well known in conventional semiconductor materials, among which the highest frequencies come from single-crystalline Si and GaAs. However, according to Supplementary Table 1, other thin-film-based candidates might not be able to exceed fifth-generation (5G) compatible frequencies higher than 20 GHz. We believe that our semitransparent WSe_2_ Schottky diode would find breakthroughs in perspectives of future 5G communications and automobile electronics toward high frequency (K-band: 18–27 GHz) applications^32,33^.

## Results

### Electrical properties of vertical Schottky diode circuit

**Fig. 1:**
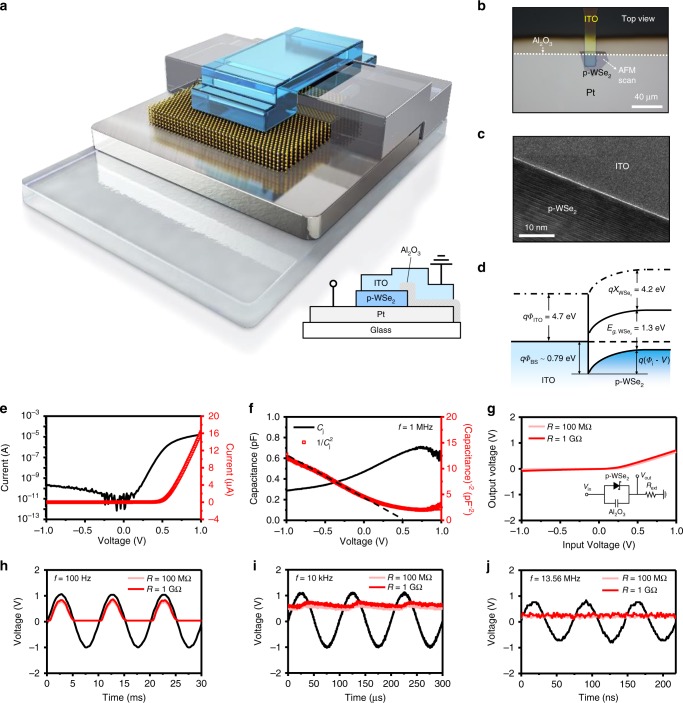
Device images and DC/AC characteristics of ITO/p-WSe_2_ Schottky diode. 3D device schematic diagram along with 2D device cross section (inset) (**a**) and OM image (**b**) of a vertical ITO/p-WSe_2_ Schottky diode. Cross-sectional TEM image (**c**) and band diagram (**d**) of ITO/p-WSe_2_ Schottky junction. *I*–*V* (**e**) and *C*_*j*_–*V* or 1/*C*_*j*_^2^–*V* (**f**) characteristics of the ITO/p-WSe_2_ Schottky diode through which WSe_2_ thickness could be worked out to be 50.5 nm, which appears well matched to the results from AFM thickness profiles (Supplementary Fig. 6). **g** Static *V*_in_–*V*_out_ curve was obtained by sweeping *V*_in_ from −1 to +1 V, along with the inset device circuit where external resistance of 100 MΩ and 1 GΩ is used. Observation of half-wave rectification in *V*_in_ (black)–*V*_out_ (red) plots obtained from the diode circuit with a parallel capacitor at 100 Hz (**h**), 10 kHz (**i**), and 13.56 MHz (**j**).

Figure 1a, b shows a three-dimensional (3D) device schematic diagram and an optical micrographic (OM) image of ITO/p-WSe_2_ Schottky diode, which has a relatively thin Al_2_O_3_ as a parallel capacitor (Type A). Depending on the overlap area of bottom Pt electrode and Al_2_O_3_, we categorize devices into Type A (large overlap area) and Type B (small overlap area). All the device fabrication processes including atomic layer deposition (ALD) of 50 nm-thick Al_2_O_3_ are shown in Supplementary Fig. 1. Transmission electron microscopy (TEM) imaging and energy dispersive X-ray spectroscopic (EDS) mapping at the ITO/p-WSe_2_ junction show a very thin (0.7 nm) uniform interfacial layer as shown in Fig. 1c and Supplementary Fig. 2. Raman spectroscopy as shown in Supplementary Fig. 3 confirms the multilayer WSe_2_^34^. The energy band diagram of ITO/p-WSe_2_ junction is estimated along with the Schottky barrier height as shown in Fig. 1d. For the Schottky barrier height and energy band estimation, temperature-dependent current density–voltage (*J*–*V*) measurements and Richardson’s equation have been utilized as shown in Supplementary Fig. 4^35–37^. The work function of the bottom Pt electrode matches well with the valence band edge of p-WSe_2_, which provides an excellent Ohmic contact to p-WSe_2_. The current–voltage (*I*–*V*) characteristics of the Type A Schottky diode show a high ON/OFF current ratio of ~10^5^ and an ideality factor (*η*) of 1.23 (Fig. 1e). Some of non-ideality (*η* > 1) may come from possible junction defects at the ITO/p-WSe_2_ interface, as conjectured from TEM observation. The detailed characteristics of Schottky diodes using different WSe_2_ thicknesses are shown in Supplementary Fig. 5^22^. Figure 1f shows the junction capacitance–voltage (*C*_*j*_–*V* or 1/*C*_*j*_^2^–*V*) characteristics for our Schottky diode. Both the *C*_*j*_–*V* measurements and the atomic force microscopy (AFM) imaging give consistent thickness values of WSe_2_ flake, 50.4 nm (*C*_*j*_–*V*) and 55.2 nm (AFM) (Supplementary Fig. 6). The full depletion thickness (*X*_*d*_) of the diodes under reverse bias would approach to the physical thickness of WSe_2_ flake, and it is estimated from saturated *C*_*j*_ value (= *ε*_WSe2_*A*_*j*_/*X*_*d*_; *ε*_WSe2_ is the dielectric constant of 7.9*ε*_o_ and *A*_*j*_ is the junction area of 2.02 × 10^–6^ cm^2^)^38–40^. Here, *C*_*j*_ (= 0.292 pF) is extracted out by removing parallel Al_2_O_3_ capacitance (*C*_ox_ is the oxide capacitance of 0.456 pF and *A*_ox_ is the oxide area of 2.95 × 10^−^^6^ cm^2^) from the raw total *C*–*V* curve results (*C*_tot_ = 0.748 pF). Based on these *C*_*j*_–*V* results, we could even estimate the hole density (~10^17^ cm^−3^) in the thick p-WSe_2_ flake using the slope of 1/*C*_*j*_^2^–*V* plot. From the 1/*C*_*j*_^2^–*V* plot, built-in potential can also be found to be 0.514 V.

ITO/p-WSe_2_ Schottky diode with a parallel capacitor can be used for signal rectification up to megahertz (MHz) frequency range. Figure 1g shows the input voltage–output voltage (*V*_in_–*V*_out_) plots obtained from the inset circuit with the different external load resistors of 100 MΩ and 1 GΩ. According to Fig. 1h, under 100 Hz 1 V amplitude alternating current (AC) input, the dynamic rectification curve exactly shows the half-wave rectification output voltage whose peak value should be related to the plot in Fig. 1g. However, at 1 kHz, resistive–capacitive delay in output voltage signal is clearly shown as coupled with the parallel capacitance (Supplementary Fig. 7b), and almost perfect direct current (DC) output voltage of 0.6 V is observed at 10 kHz (Fig. 1i)^41^. Decreased DC output level at higher frequencies (Supplementary Fig. 7d, e) is not an intrinsic device property but rather due to an inductive impedance *ωL*_ext_ (*L*_ext_ is external inductance of coaxial cable)^42^. Figure 1j shows such decreased DC output voltage at 13.56 MHz, which is a well-known standard radio frequency (RF). At 13.56 MHz, DC output voltage appears to be as small as 0.2 V, but it is enough to be used for demodulation of AM radio signals.

### AM demodulation for radio reception

**Fig. 2:**
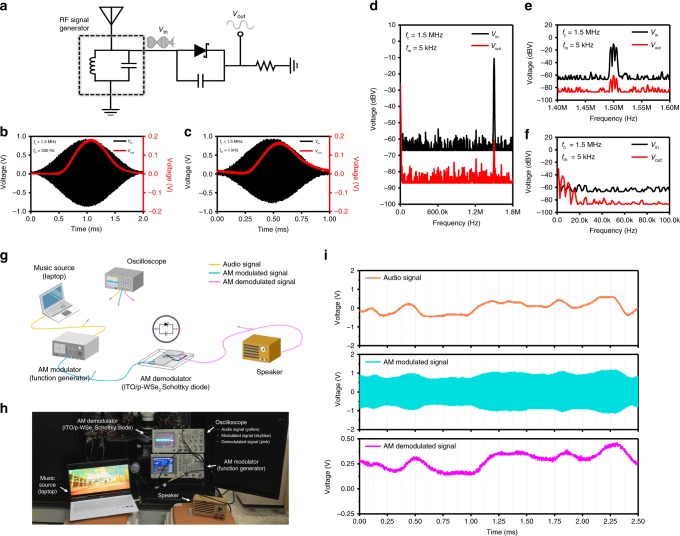
AM radio receiver. **a** Schematic circuit diagram of AM radio receiver including an antenna. Amplitude modulated input (black) and demodulated output (red) voltage signals in time domain spectra as obtained at *f*_*c*_ = 1.5 MHz with *f*_*m*_ = 500 Hz (**b**) and 1 kHz (**c**). **d** Fast fourier transform (FFT) input (black) and output (red) spectra in frequency domain at *f*_*c*_ = 1.5 MHz with *f*_*m*_ = 5 kHz. **e** Zoomed FFT spectra for symmetric baseband which consists of three fine peaks at 1.5 MHz and ±5 kHz apart from the central 1.5 MHz (carrier feed-through signal peak). They also show much reduced AM input, which indicates demodulation effects. **f** Zoomed FFT spectra near 5 kHz, showing main peaks of demodulated signals at 0 Hz and 5 kHz along with distortion-induced small signals at 10 and 15 kHz. Schematic illustration (**g**) and photograph (**h**) of practical AM demodulator setup to test our Schottky device performance as composed of audio source (laptop computer), three channel oscilloscope, AM modulator (function generator), AM demodulator (ITO/p-WSe_2_ Schottky diode circuit), and speaker. **i** Three different profiles from real audio (yellow), AM modulating function generator (sky blue), and demodulation (pink) signals. Note that the demodulated signals quite resemble those of real audio as an envelope of AM signals. Supplementary Fig. 10 would explain more.

We demonstrate successful demodulation of AM radio signals using the Schottky diode. The dashed square part including antenna was simulated by using a function generator, and the Schottky diode integrated with a parallel capacitor and an external series resistor served as the main components of the demodulator (Fig. 2a). Carrier frequencies (*f*_*c*_) of 500 kHz, 1 MHz, and 1.5 MHz and the low modulating frequencies (*f*_*m*_) of 0.5, 1, and 5 kHz were selected for audio signal frequencies at a modulation index of 100%^43^. Figure 2b, c presents the time domain signals of AM input voltage (black band) overlaid with demodulated DC output voltage signals (red line with 0.17 V peak) that, respectively, result from two cases of frequency mixing [(*f*_*m*_, *f*_*c*_) = (0.5 kHz, 1.5 MHz) and (1 kHz, 1.5 MHz)]. AM input voltage signals appear well demodulated in overall view with a slight phase shift for 1 kHz signal as shown in Fig. 2c. The 1.5 MHz AM input voltage band appears just black and unresolved in our display due to too many AC oscillations in a band (but such an input band could be resolved by reducing the time frame as shown in Supplementary Fig. 8). Figure 2d shows the frequency domain signals of AM input (black signal; 5 kHz and 1.5 MHz) and demodulated output (red signal). Unlike the input showing large voltage signals near 1.5 MHz, the output shows at least two orders of magnitude reduced voltage signals at 1.5 MHz, while it also presents large demodulated voltage signals near 5 kHz. This indicates that the demodulation by our device is very effective. Figure 2e, f presents the zoomed views of signals near 1.5 MHz and 5 kHz, respectively (see more details in Supplementary Fig. 9). The demodulation of real audio signals carried at 1.5 MHz was tested as shown in Fig. 2g, h. The oscilloscope-captured signals clearly display the three different profiles from real audio, AM modulating function generator, and demodulation signals (Fig. 2i). The demodulated signals resemble those of real audio in profile with very slight distortion, and even a Korean pop music was successfully demodulated (see the experimental setup in Supplementary Fig. 10 for more details)^44^.

### Gigahertz frequency rectification behavior

**Fig. 3:**
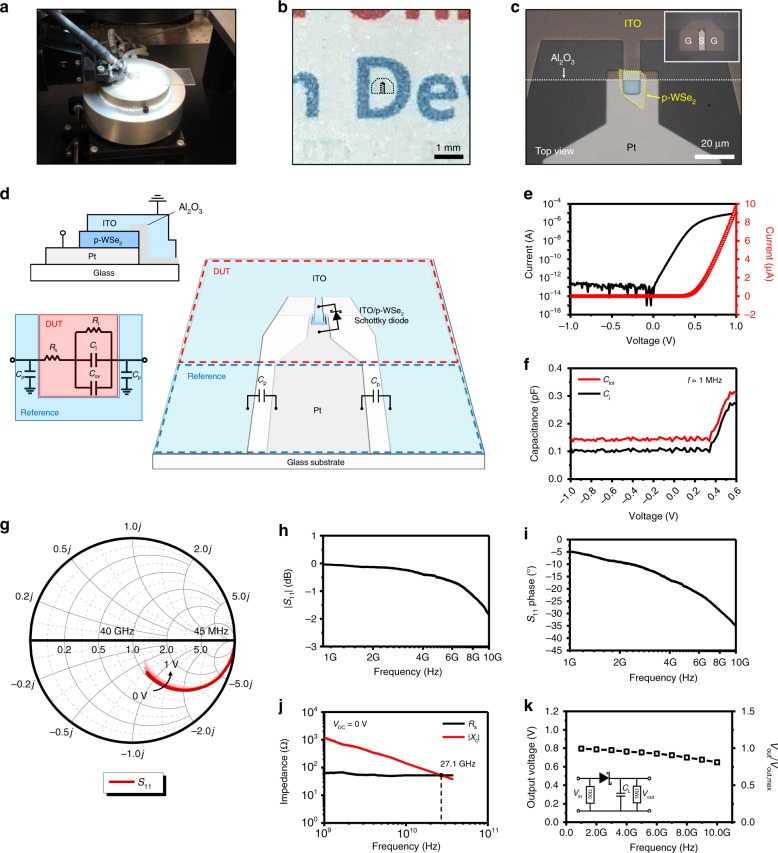
Semitransparent ITO/p-WSe_2_ Schottky device architecture for RF measurements. Photograph of RF probing (**a**), OM image of our device showing semitransparency on a background paper (**b**), magnified OM image without background paper (**c**), and CPW configuration (**d**) of the Schottky diode prepared on the G-S-G pattern. The inset OM image of **c** is a zoomed image of the device in **b**. The top and bottom insets of **d** are a 2D cross-section schematic and an equivalent small signal model of the Type B diode for the high frequency RF measurement. *R*_*s*_, *R*_*j*_, *C*_*j*_, and *C*_ox_ are noted in the circuit model. The parasitic capacitance (*C*_*p*_) can be negligible compared with *C*_ox_ and *C*_*j*_. *I*–*V* (**e**) and *C*–*V* (**f**) characteristics of the ITO/p-WSe_2_ Schottky diode. Both of approximate *C*_*j*_ and *C*_tot_ (= *C*_*j*_+*C*_ox_) with parallel Al_2_O_3_ are displayed. **g** Smith chart from 45 MHz to 40 GHz under forward DC biases (0 to +1 V) as obtained from *S*_11_ parameter measurement. As a positive voltage is applied, the red curve goes inward. Magnitude (**h**) and phase angle (**i**) plots of *S*_11_ parameters in the Schottky device. **j** *R*_*s*_ and |*X*_*c*_|(= 1/*ωC*_tot_) plots as a function of frequency at 0 V (battery free). The cutoff frequency is found at 27.1 GHz. The plots are without de-embedding. **k** Average rectified *V*_out_ as a function of frequency of the input AC signal with an amplitude of 1 V. Inset circuit is for rectified DC output voltage measurements. According to **h**, **j**, and **k**, cutoff frequency should be much higher than 10 GHz and approaches to ~27 GHz.

With intrinsically high cutoff frequencies of our ITO/WSe_2_ Schottky diodes, even successful gigahertz (GHz) rectification can be demonstrated. RF probing was performed from 45 MHz to 40 GHz for one-port scattering (*S*_11_) parameter measurements on our Schottky diodes as seen in the photograph of Fig. 3a. For the *S*_11_ parameter measurement, the Schottky diode (Type B) was prepared on the mesa structure pattern with 100 μm ground-signal-ground (G-S-G) pitch. The OM images of Fig. 3b, c show semitransparency and zoomed details of the device, respectively. Coplanar waveguide (CPW) configuration is shown in the inset of Fig. 3c and Fig. 3d, as fabricated with a characteristic impedance which is close to 50 Ω based on the dielectric constant of glass and dimension of metal pads^45,46^. In the CPW pattern of the Type B diode (the top inset in Fig. 3d), the junction area is reduced to 0.69 × 10^−6^ cm^2^ which is ~3 times smaller than the Type A diode, while the parallel oxide-capacitive area (0.26 × 10^−^^6^ cm^2^) is an order of magnitude (or 12 times) smaller (the inset of Fig. 1a). Figure 3e shows the *I*–*V* characteristics of the Type B diode, showing much improved DC properties compared with those of the Type A device: three orders of magnitude lower reverse leakage current (110 fA) and two order of magnitude higher ON/OFF ratio (~10^7^) along with an improved ideality factor of 1.12. All of these positive effects should be related to the reduced junction and oxide-capacitive area. Moreover, such a reduced junction area leads to smaller junction capacitance (0.10–0.12 pF) as well as the total capacitance. The *C*–*V* characteristics in Fig. 3f show the total capacitance (0.15 pF) with 48 nm-thick WSe_2_ in Supplementary Fig. 11, which is five times smaller than that (0.75 pF) of the Type A diode with similar thickness (55 nm) of WSe_2_. The Type B Schottky diodes with reduced junction and oxide-capacitive area are thus expected to achieve a superior cutoff frequency performance in RF measurements.

Figure 3g shows that the Smith chart results from the Schottky diode as measured from 45 MHz to 40 GHz under different DC forward biases from 0 to +1 V. The bottom inset of Fig. 3d is an equivalent small signal circuit model of high frequency RF measurements on device under test (DUT) structure. Since Al_2_O_3_ is deposited on WSe_2_ flake rather than Pt electrode, oxide-induced capacitance (*C*_ox_) is parallel to junction resistance (*R*_*j*_) and junction capacitance (*C*_*j*_)^22^. At a sufficiently high frequency, the *C*_*j*_ dominates the *R*_*j*_. The resultant total resistance is series resistance (*R*_*s*_) which is now mostly covered by contact resistance (*R*_*c*_), because the *R*_*j*_ becomes negligible at high frequency. Accordingly, a useful “figure of merit” would be the frequency at which total capacitive reactance (|*X*_*c*_| = 1/*ωC*_tot_ where *C*_tot_ = *C*_*j*_ + *C*_ox_; *C*_ox_ would be <40 fF) equals to *R*_*s*_, and it is called the cutoff frequency (*f*_cutoff_) as it also matched to the −3 dB power loss point^47–49^. Although there must exist the effects of parasitic components for the 48.3 nm-thick WSe_2_ Schottky diode, the diode well displays *S*_11_ plots for magnitude and phase angle in Fig. 3h and i, respectively. According to Fig. 3h, −3 dB power loss is expected at much higher than 10 GHz. Figure 3j shows the value of *R*_*s*_ and |*X*_*c*_|(= 1/*ωC*_tot_) extracted from *S*_11_ results at zero DC bias (*V*_DC_ = 0 V) as a function of frequency. It is expected from the plots that the diode with 48.3 nm-thick WSe_2_ has a cutoff frequency of over 27 GHz, and such a high cutoff frequency stems from extremely low *R*_*s*_ value (~50 Ω), mostly of contact resistance. As the frequency of the input signal increases, the value of the rectified output signal slightly decreases from *V*_out_ maximum (0.80 V) but is still maintained to be 0.65 V (*V*_out_/*V*_out,max_ = ~81%) at 10 GHz as shown in Fig. 3k^50–53^. Such voltage rectification at even higher frequencies has been again confirmed from two-port Schottky device architecture, according to which the *f*_cutoff_ is observed at ~25 GHz with a similar WSe_2_ thickness of ~50 nm (Supplementary Fig. 12).

**Fig. 4:**
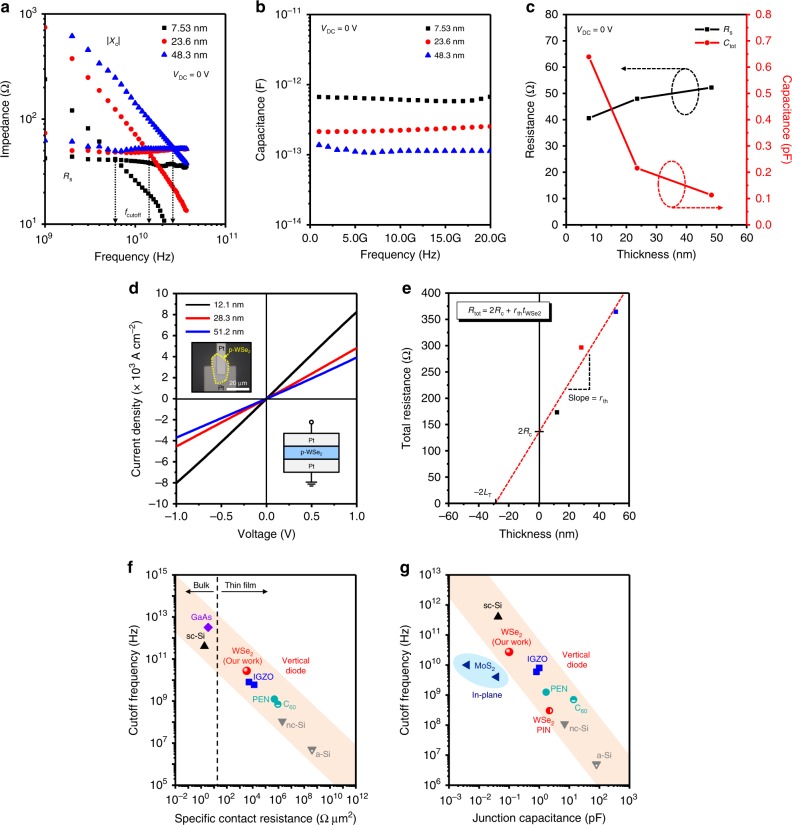
WSe_2_ thickness-dependent cutoff frequency behavior and extremely small contact resistance. **a** *R*_*s*_ and |*X*_*c*_|(= 1/*ωC*_tot_) plots as a function of frequency under zero external bias (battery free). The *f*_cutoff_ is found at 27.1, 14.7, and 5.92 GHz for 48.3, 23.6, and 7.53 nm-thick WSe_2_ when plotted without de-embedding. **b** Thickness-dependent capacitance values extracted from |*X*_*c*_|(= 1/*ωC*_tot_) plots. **c** *R*_*s*_ and *C*_tot_ plots as a function of WSe_2_ thickness. Unlike capacitance, *R*_*s*_ does not vary in the range between 40 and 52 Ω. **d** *J*–*V* characteristics of the Pt-WSe_2_-Pt diodes (inset) with different WSe_2_ thicknesses of 12.1, 28.3, and 51.2 nm. They show straight ohmic behavior. **e** Total resistance-thickness (*R*_tot_−*t*_WSe2_) plot extracted from *J*–*V* plots based on the same contact area (*A*_ct_ = 0.69 × 10^−^^6^ cm^2^) of the G-S-G diodes. The red dashed line represents a linear fitted line for the three *R*_tot_ points with different *t*_WSe2_. *R*_*c*_ is worked out to be 65 Ω (*L*_*T*_ = 28.2 nm, *W* = 8.3 μm). This *R*_*c*_ value is almost the same as that of *R*_*s*_ because *R*_*s*_ is mostly covered by *R*_*c*_ in high frequency RF measurement, and in fact should be constant regardless of measurement type. Figure of merit comparison in the plot of *R*_ct_ vs. *f*_cutoff_ (**f**) and in the other plot of *C*_*j*_ vs. *f*_cutoff_ (**g**). In view of **f** and **g**, our *f*_cutoff_ utilizing 3400 Ω μm^2^ (= 50 Ω × 69 μm^2^) and 0.10 pF appears quite outstanding, showing the best RF performance if those of bulk single-crystalline Si- and GaAs-based Schottky diodes are excluded. These results indicate that contact resistance and junction capacitance are dominating factors for high frequency operation.

The thickness of WSe_2_ can be utilized to modulate the cutoff frequency of the Schottky diode effectively. Figure 4a displays cutoff frequency (Type B) with different WSe_2_ thicknesses of 7.53, 23.6, and 48.3 nm. Interestingly, the Schottky diodes incorporating thinner WSe_2_ (7.53 and 23.6 nm) show lower cutoff frequencies at 5.92 and 14.7 GHz, respectively. The *C*_tot_ extracted from |*X*_*c*_|(= 1/*ωC*_tot_) plot of Fig. 4a shows the generally inversely proportional value with respect to the thickness of WSe_2_ (Fig. 4b, c). On the other hand, the *R*_*s*_ values show nearly constant values around ~50 Ω regardless of WSe_2_ thickness (Fig. 4c). The *f*_cutoff_ equation below genuinely explains the above thickness-dependent results, but we also calculated *f*_cutoff_ following Eq. (1) and confirmed that calculation results are almost the same as the measured value in each thickness^22,49^:

1 $$f_{{\mathrm{cutoff}}} = \frac{1}{{2{\uppi}R_{s}\left( {C_{j} + C_{{\mathrm{ox}}}} \right)}}.$$

The thickness-dependent *f*_cutoff_ results signify that *f*_cutoff_ even higher than 27 GHz is possibly achievable by optimally modulating the WSe_2_ thickness. More details on each DUT Schottky device of 27.1, 14.7, and 5.92 GHz are displayed in Supplementary Figs. 13–15.

The observed ultra-low *R*_*s*_ of ~50 Ω, independent of WSe_2_ thickness in high frequency regime, mainly originates from the *R*_*c*_, which must be constant for either RF or DC measurement. In order to confirm such a low *R*_*c*_, we conducted DC regime *J*–*V* characteristics on Pt-WSe_2_-Pt diode with three different thicknesses of WSe_2_. This experiment could be regarded as a modified transmission line measurement because it suitably provides *R*_*c*_^54^. Figure 4d displays the *J*–*V* characteristics of the Pt-WSe_2_-Pt diodes with different WSe_2_ thicknesses of 12.1, 28.3, and 51.2 nm. Those *J*–*V* curves show straight ohmic behavior, and *R*_*c*_ is estimated to be 65 Ω, which is quite similar to *R*_*s*_ in RF measurements (Fig. 4e). Extremely small specific contact resistance, *R*_ct_ (= *R*_*c*_  × *A*_ct_) is thus estimated to be 3.4–4.2 × 10^−5^ Ω cm^2^ (*A*_ct_ is the contact area of 0.69 × 10^−^^6^ cm^2^ and *L*_*T*_ is the characteristic transfer length of 28.2 nm as measured from *x*-axis intercept). The measured contact resistance of 50–65 Ω is a record-low *R*_*c*_ value for 2D layered semiconducting crystals, to the best of our limited knowledge. According to Fig. 4f, g and Supplementary Table 1, the highest *f*_cutoff_ is found to be over 1 THz and 400 GHz, which are, respectively, from vertical GaAs- and Si-based bulk single-crystalline Schottky diodes, since *R*_ct_ values of those devices are extremely low (10^−^^7^–10^−^^8^ Ω cm^2^) and the devices are fabricated with low capacitances^55,56^. Our *f*_cutoff_ value (27.1 GHz) is located right after them, showing that ours is still outstanding among all other thin-film semiconductors. Out-of-plane linear mobility (*μ*) of 2.2 cm^2^ V^−^^1^ s^−^^1^ is also extracted from the slope (*r*_th_ = 1/*pqμA*_ct_) of 4.8 Ω nm^−1^, where hole concentration (*p*) in WSe_2_ is assumed to be ~10^17^ cm^−3^ from Supplementary Fig. 11^40^.

### Ultraviolet–visible photodetection

Our ITO/p-WSe_2_ diodes are equipped with a transparent electrode window and can be functional under visible photons. According to the photocurrent–voltage (*I*_ph_–*V*) and responsivity plots shown in Supplementary Fig. 16, the photoelectric and photovoltaic properties of ITO/p-WSe_2_ Schottky diode are well noted in the visible range^57,58^.

## Discussion

In summary, our Schottky diodes incorporating rather thick WSe_2_ provide an alternative approach to utilize layered van der Waals semiconductors for device applications. Our results show that the cutoff frequency of Schottky diodes can be increased as we incorporate thicker 2D layered crystals. The reliable excellent Schottky junction and Ohmic contact to WSe_2_ were successfully achieved using conventional device fabrication processes compatible with semiconductor industry. The demonstrated 27.1 GHz cutoff frequency is matched with 5G era wireless communication as the highest among cutoff frequency reports from 2D material- or 3D thin-film-based diodes (Supplementary Table 1). Conventional thin-film deposition techniques could also be employed to grow quite thick large-area WSe_2_ or other TMDs^59,60^. Controlling the density of defects, the grain size, and the thickness of WSe_2_ from thin-film growth would be the key requirement for our high frequency applications. Considering the demonstrated cutoff frequency of 27.1 GHz with exfoliated samples is approximately an upper limit, we believe that the device performance with large-area samples could be good enough for at least a few GHz applications. We thus conclude that our vertical semitransparent Schottky diode would be a breakthrough device toward ubiquitous 5G wireless communications and automobile electronics while serving as a key component built in window glass.

## Methods

### Device fabrication

A glass substrate (Eagle XG) was ultrasonically cleaned by acetone, ethanol, and deionized water and then dried by N_2_ gas flow. A Pt bottom electrode (10 nm) was patterned with photolithography and deposited on the glass substrate by DC magnetron sputtering. Thick p-type WSe_2_ flakes (HQ Graphene) were mechanically exfoliated and transferred onto the Pt electrode. For the ITO/p-WSe_2_ Schottky diode, the device was annealed in air ambient at 300 °C for 10 min. A 50 nm-thick high-k dielectric Al_2_O_3_ was patterned by conventional photolithography and deposited by the ALD system at the edge of the WSe_2_ flake. Then, an ITO top electrode (100 nm) was patterned by the photolithography and deposited by DC magnetron sputtering on the side where Al_2_O_3_ was deposited. Besides Schottky diodes, Pt-WSe_2_-Pt diodes were also fabricated, to obtain the information on *R*_*c*_, as follows. First, a Pt bottom electrode (10 nm) was patterned with the photolithography and deposited on the glass substrate by DC magnetron sputtering. The WSe_2_ flakes with different thicknesses were mechanically exfoliated and transferred onto the bottom electrode. Then, a Pt top electrode (100 nm) was patterned by the photolithography and deposited by DC magnetron sputtering on the WSe_2_ flake. In air ambient, the device was annealed at 300 °C for 10 min. For DUT structure in RF measurements which use the characteristic impedance in transmission lines with 50 Ω for RF matching, the width of the signal metal pad was designed to be 80 μm, and the distance between the signal and ground pad was designed as 13 μm, based on glass substrate with a dielectric constant (5.181) and thickness (0.5 mm). Our devices were mostly reproducible in their fabrication and DC performances when at least four batches were measured for 32 device samples in total. Four batches are categorized by WSe_2_ thickness: ~10, ~30, ~50, and ~70 nm. All the samples endured more than a month without visible property degradation in air ambient. More details are found in Supplementary Fig. 5.

### Materials characterization

AFM equipment (XE-100, park systems) was used to measure the WSe_2_ thickness, and Raman spectroscopy (LabRam Aramis, Horriba Jovin Yvon) was performed by using 532 nm laser source.

### TEM characterization

All of the cross-sectional TEM samples were fabricated with an FEI Helios 650 dual beam focused ion beam. TEM imaging and EDS mapping were performed with transmission electron microscopes (JEOL JEM-F200 and 2100-plus), operated at 200 kV.

### Electrical and photoelectrical measurement

Electrical and photoelectrical measurements were performed by using a semiconductor parameter analyzer (HP4155C, Agilent Technologies). Low-temperature electrical measurement was carried out in the dark at vacuum (~1.5 mTorr) in the temperature range from 180 to 320 K at 20 K intervals. Photoelectrical measurement was performed in the dark and under LED illumination: near-infrared (950 nm, 4.5 mW cm^−^^2^; 850 nm, 13.2 mW cm^−2^), red (620 nm, 2.3 mW cm^−2^), green (520 nm, 2.2 mW cm^−2^), blue (470 nm, 1.1 mW cm^−2^), and ultraviolet (400 nm, 3.9 mW cm^−2^). Capacitance measurements are measured by using a precision LCR meter (HP4284A, Agilent Technologies) with a small signal frequency of 1 MHz.

### Half-wave rectification and AM demodulation measurement

All AC input voltage signals were applied by increasing ten units from 100 Hz to 20 MHz under the conditions that the peak voltage was +1 V, and the offset was 0 V with a function generator (AFG 3022B, Tektronix). The AC input voltage and rectified output voltage were measured with a four channel digital storage oscilloscope (TDS 2014B, Tektronix). In the same conditions of the peak voltage and offset, amplitude modulated input voltage signals were applied with 1.5 MHz carrier frequency and signal/modulating frequencies of 0.5, 1, and 5 kHz, respectively, at 100% modulation index. The input amplitude modulated voltage and output demodulated voltage were measured by the same oscilloscope.

### Cutoff frequency measurement

One-port scattering (*S*_11_) parameter measurements were performed by a vector network analyzer (VNA; E8364A, Agilent Technologies) after calibration. The RF source was set to sweep from 45 MHz to 40 GHz with 12,800 interval points, and an internal reflectometer was used to detect the incident and reflected RF wave signals. The magnitude and phase of *S*_11_ were measured and recorded. *S*_11_ represents the reflection coefficient, which is the ratio of reflected signal voltage to the incident signal voltage at one single port. The real part and imaginary part of impedance are calculated by *S*_11_ = (*Z*_11_ − *Z*_0_)/(*Z*_11_ + *Z*_0_), where *Z*_0_ = 50 Ω. The *S*_11_ parameter measurements were repeated under different applied forward bias voltages from 0 to +1 V. For rectified *V*_out_ measurement, AC input signal of 1 V was generated by a signal generator (N5183A, Agilent Technologies) so that the rectified DC output signal was measured by an oscilloscope (DSO81004B, Agilent Technologies) with impedance of 1 MΩ.

## Supplementary information

Supplementary Information

## Data Availability

The authors confirm that the data supporting the findings of this study are available within the article and its supplementary materials from the corresponding authors upon reasonable request.
