# Supplementary material for: Ultrafast 27 GHz cutoff frequency in vertical WSe2 Schottky diodes with extremely low contact resistance
Source: Nat Commun. 2020 Mar 27;11:1574. doi: 10.1038/s41467-020-15419-1 (PMC7101435; doi:10.1038/s41467-020-15419-1)
Supplement: Supplementary file 1 — Supplementary Information [file 41467_2020_15419_MOESM1_ESM.pdf]

Supplementary Information

Ultrafast 27 GHz cutoff frequency in vertical WSe_2 Schottky diodes with extremely low contact resistance

Yang et al.

Supplementary Figures

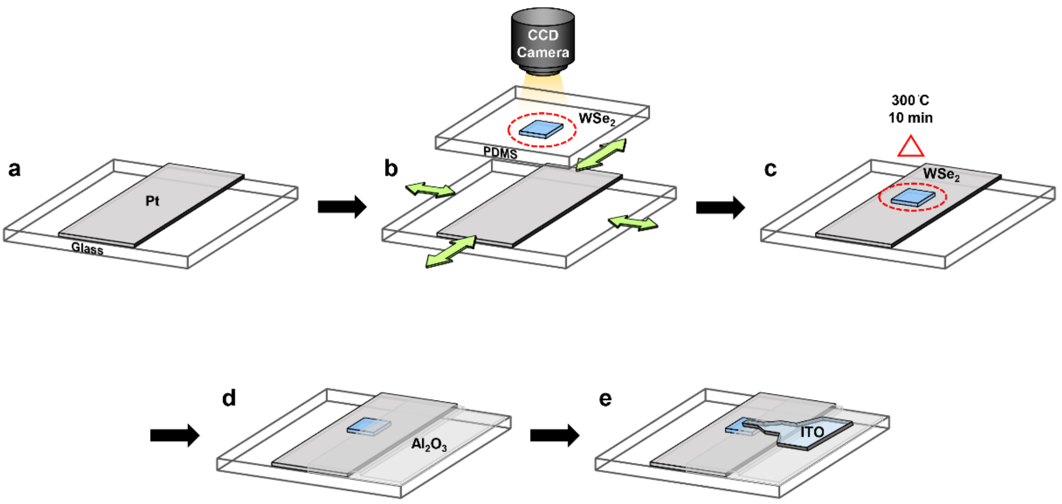

Supplementary Figure 1 Fabrication procedure of ITO/p-WSe_2 Schottky diode. **a** Photolithographic patterning and depositing of Pt bottom electrode on glass substrate. **b** Dry transfer of p-WSe_2 flake onto the Pt electrode. **c** Contact annealing in air ambient at 300 ˚C for 10 minutes. **d** Patterning and depositing of Al_2O_3 by atomic layer deposition (ALD) system at the edge of p-WSe_2 flake. **e** Depositing and photolithographic patterning of ITO top Schottky electrode on the side of Al_2O_3.

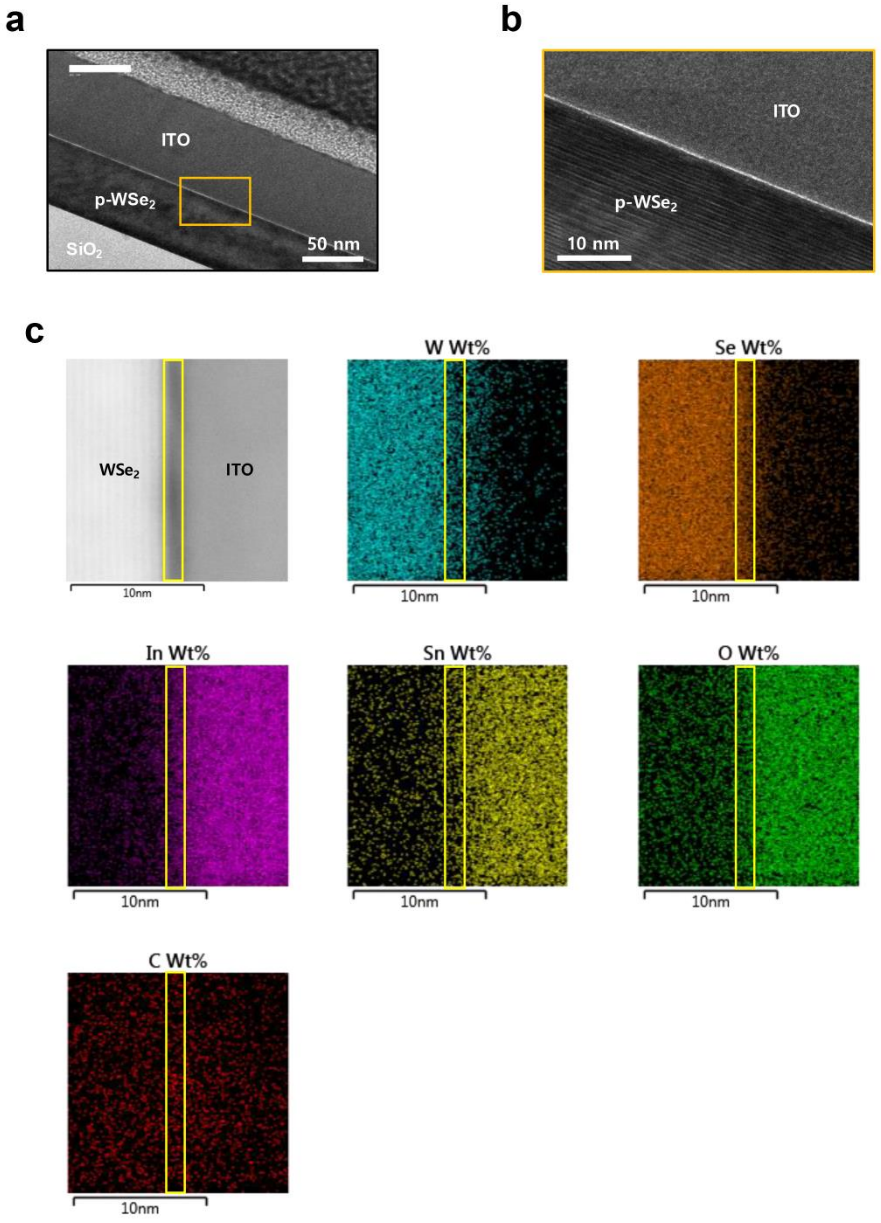

Supplementary Figure 2 ITO/p-WSe_2 junction interface. **a, b** Transmission electron microscopy (TEM) image (**a**) and the zoomed TEM image (**b**). **c** Energy dispersive spectroscopy (EDS) elemental mapping.

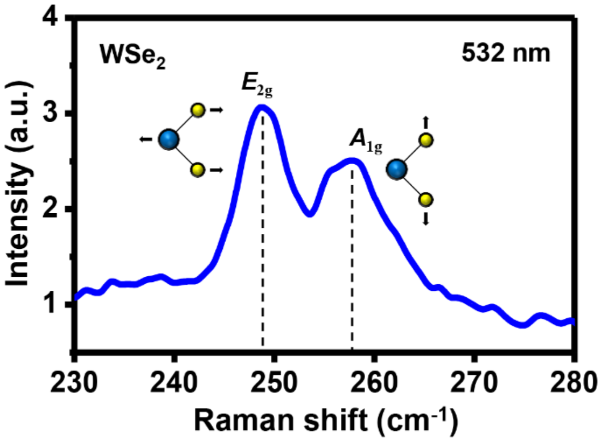

Supplementary Figure 3 Raman spectroscopy. Raman spectra measured on thick WSe_2 flake in Fig. 1b.

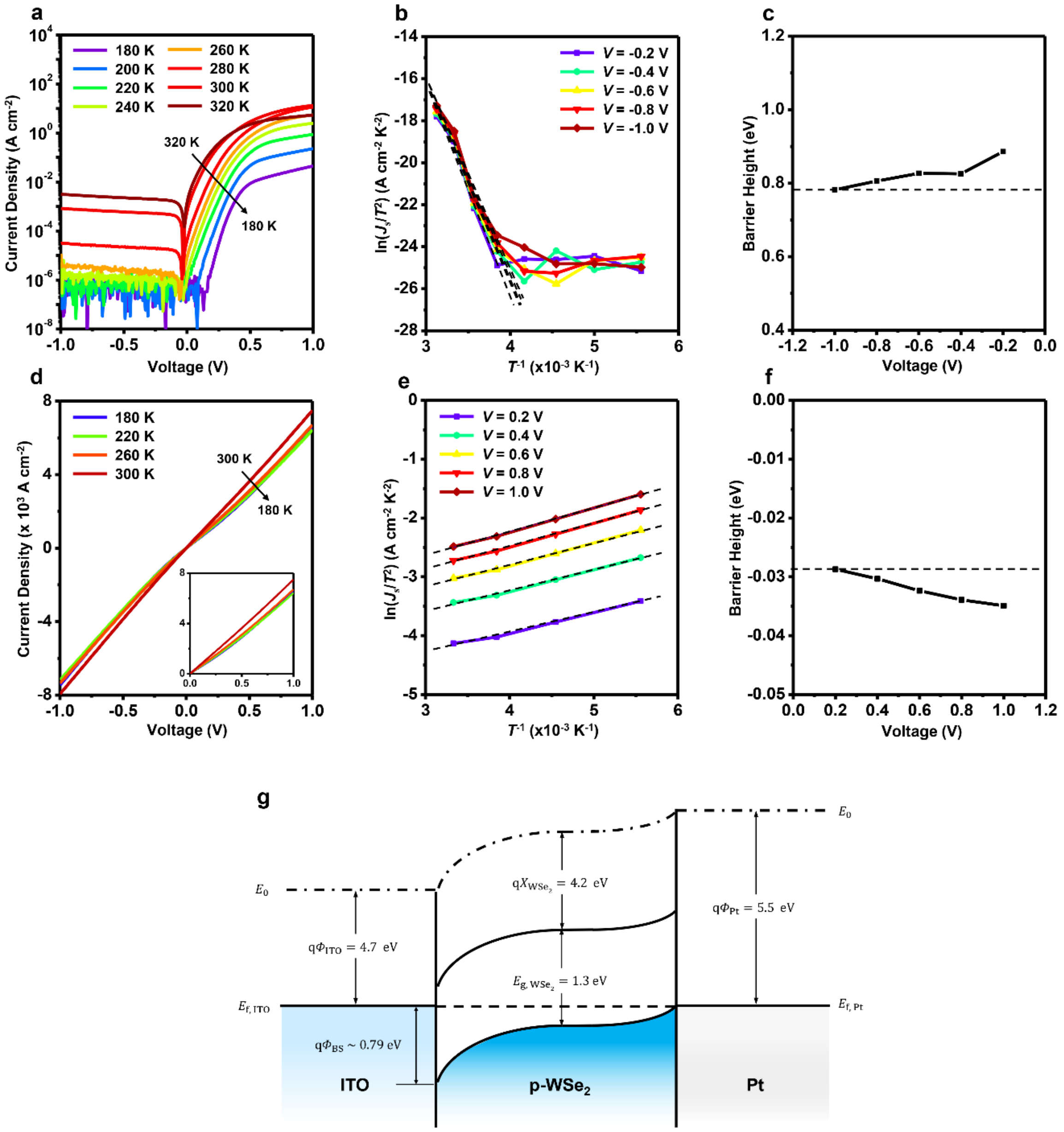

Supplementary Figure 4 Schottky barrier height extraction and energy diagram estimation of ITO-WSe_2-Pt structure. **a-c** Temperature-dependent current density-voltage (*J*-*V*) characteristics (**a**), Richardson plot (**b**), and Schottky barrier height data plot (**c**) of ITO/p-WSe_2 Schottky junction at vacuum (~1.5 mTorr). Approximate $q\Phi_{BS}$ is worked out to be ~0.79 eV. **d-f** Temperature-dependent current density-voltage (*J*-*V*) characteristics (**d**), Richardson plot

(**e**), and Schottky barrier height data plot (**f**) of Pt/p-WSe_2 Ohmic junction at vacuum (~1.5 mTorr). **g** Estimated energy diagram of ITO-WSe_2-Pt structure.

For detailed considerations on the Schottky barrier heights at both interfaces of ITO/p-WSe_2 and Pt/p-WSe_2, we conducted temperature-dependent Schottky barrier height estimation as below, following Richardson's equation, $\ln(J_s/T^2)=\ln(A^*)-q\Phi_B/k_BT$, where J_s is the saturation current density, A^* (= ~27.6 A cm^{-2} K^{-2}) is the effective Richardson constant of p-WSe_2, q is the electric charge, Φ_B is the Schottky barrier height, and k_B is the Boltzmann constant.

$$\ln\left(\frac{J_s}{T^2}\right) = \ln(A^*) - \frac{q\Phi_{BS}}{k_B T} = -\frac{q\Phi_{BS}}{1000k_B}\left(\frac{1000}{T}\right) + \ln(A^*) \quad \text{and} \quad \Phi_{BS} = 0.79\ \text{eV}$$

According to the above Richardson plot extracted from temperature-dependent *J-V* curves for ITO/p-WSe_2 Schottky junction, the $q\Phi_{BS}$ at reverse voltage (-0.5 ~ -1.0 V) is around 0.79~0.81 eV, which is consistent with the curve-fitting results ($q\Phi_{BS}$ = ~0.79 eV) in Supplementary Fig. 11e.

As for the Ohmic junction, we also fabricated Pt-WSe_2-Pt diode and tried similar measurements as seen below.

$$\ln\left(\frac{J_s}{T^2}\right) = \ln(A^*) - \frac{q\Phi_{BO}}{k_B T} = -\frac{q\Phi_{BO}}{1000k_B}\left(\frac{1000}{T}\right) + \ln(A^*) \quad \text{and} \quad \Phi_{BO} = -0.028\ \text{eV}$$

In this case, the slope in Richardson plot appears positive unlike the case of ITO/p-WSe_2 junction, although conductance decreases as the temperature decreases. The $q\Phi_{BO}$ is thus extracted to be negative but very small, ranging from -0.028 to -0.035 eV, which is as small as thermal energy at room temperature (~0.026 eV). These results simply mean that almost no Schottky barrier exists at the large area Pt/p-WSe_2 junction (or the values are in error range. Theoretically, no negative Schottky barrier height exists.). Hence, the observed extremely small contact resistance of 50 Ω is quite reasonable, and the following energy band diagram is appropriately provided as above.

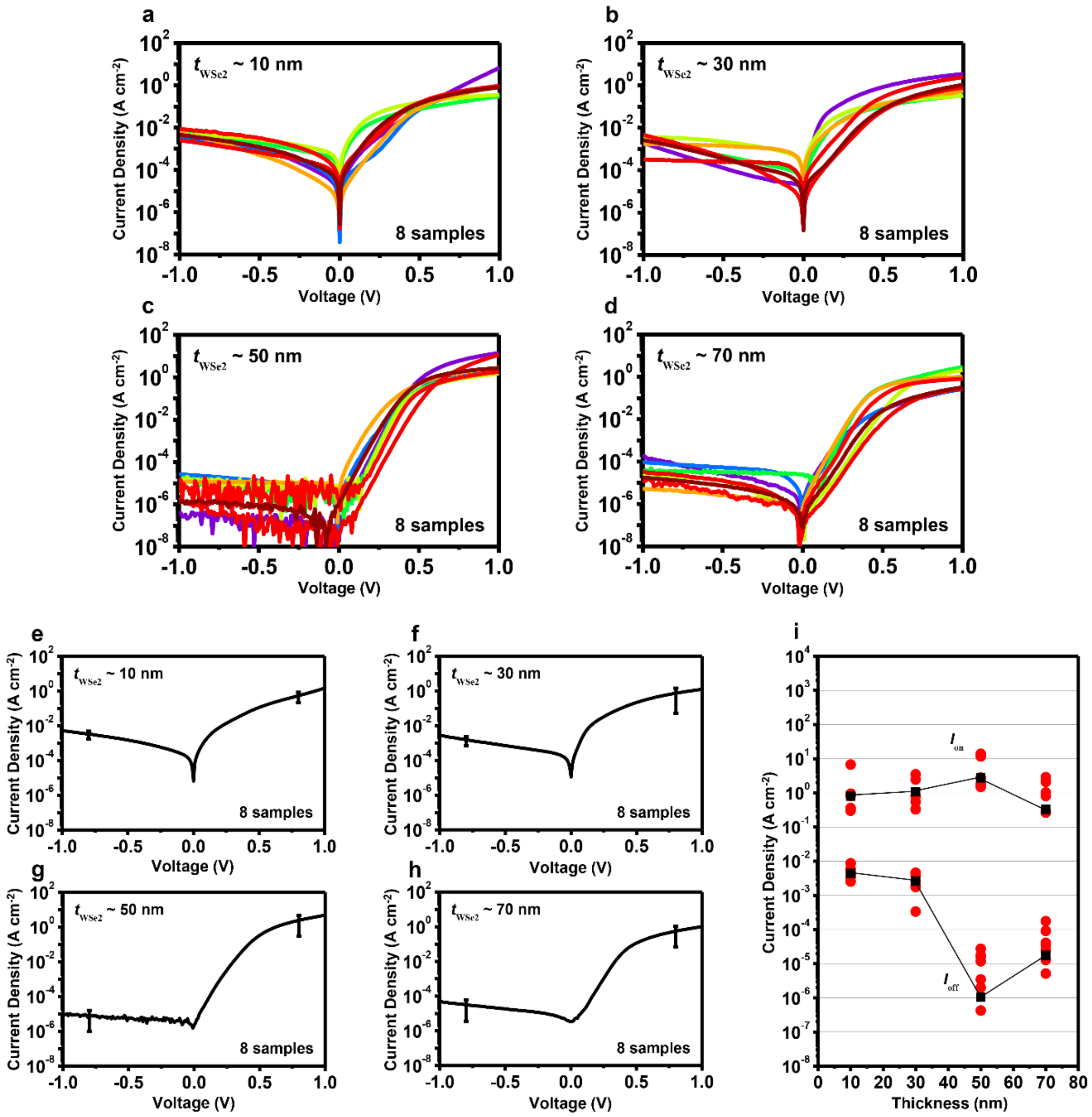

Supplementary Figure 5 Electrical characteristics of ITO/p-WSe_2 Schottky diodes with different WSe_2 thicknesses at room temperature. **a-d** A collection of current density-voltage (*J*-*V*) curves with different WSe_2 thicknesses of ~10 nm (**a**), ~30 nm (**b**), ~50 nm (**c**), and ~70 nm (**d**). **e-h** Normalized current density-voltage (J_n-*V*) curves with different WSe_2 thicknesses of ~10 nm (**e**), ~30 nm (**f**), ~50 nm (**g**), and ~70 nm (**h**). **i** Summarized data plot of ON and OFF current densities according to the thicknesses.

We prepared 4 batches of Schottky diodes according to their WSe_2 thickness: ~10 nm, ~30 nm, ~50 nm, and ~70 nm. Each batch has 8 devices. So, we have fabricated ~32 devices in total. First of all, they mostly endured more than a month at least since their device surface is covered by ITO electrode, which would protect further increase of the p-doping level. The data above shows the J-V characteristics of all the devices aged for more than a month. The best results of the ON/OFF ratio were achieved from ~50 nm thick vertical Schottky diodes. As seen above, high reverse leakage is found from devices with too thin WSe_2. The rest figures are the normalized J_n-V results along with the error bars (s.d.) at V = ±0.8 V, and the summary of ON and OFF current densities is also shown as a data plot.

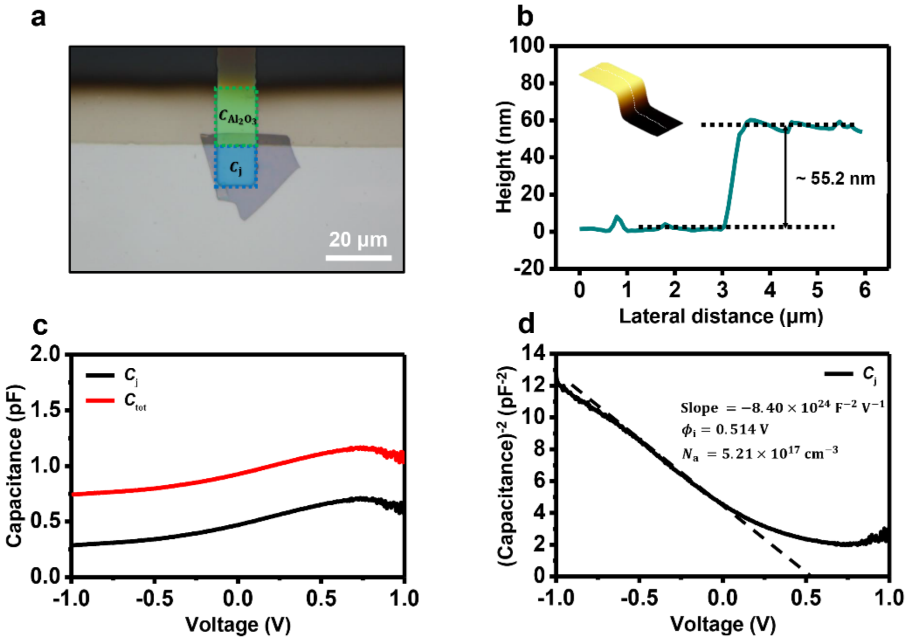

Supplementary Figure 6 Capacitance-voltage characteristics of ITO/p-WSe_2 Schottky diode for AM demodulation. **a** Optical microscopic (OM) image showing the separation of junction capacitance area (blue) and oxide capacitance area (green). **b** Atomic force microscopy (AFM) scan image/thickness profiles are shown for the WSe_2 flake in Fig.1b. **c** Capacitance-voltage (*C*-*V*) characteristics of ITO/p-WSe_2 Schottky diode with (dashed) and without (solid) parallel Al_2O_3 capacitor (C_{ox} = ~0.45 pF). **d** $1/C^2$-V characteristics of ITO/p-WSe_2 Schottky diode without (solid) parallel Al_2O_3 capacitor show approximate hole concentration N_a, 5.27x10^{17} cm^{-3}.

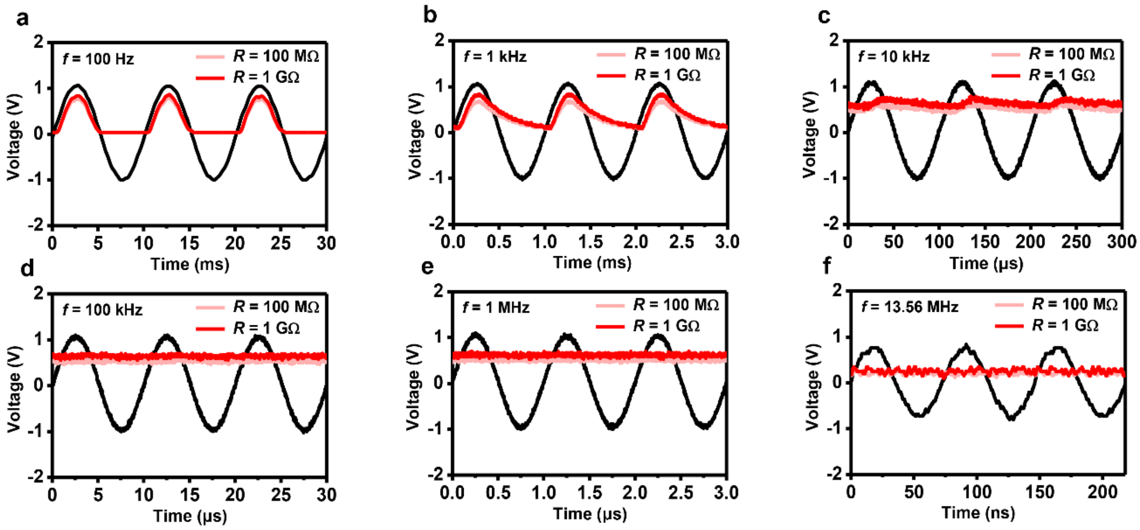

Supplementary Figure 7 Half-wave rectifying characteristics of ITO/p-WSe_2 Schottky diode with a parallel capacitor. **a-f** Half-wave rectification in V_{in} (black)-V_{out} (red) signals obtained from the diode circuit with a parallel capacitor at 100 Hz (**a**), 1 kHz (**b**), 10 kHz (**c**), 100 kHz (**d**), 1 MHz (**e**), and 13.56 MHz (**f**). Half-wave rectification in V_{in} (black)-V_{out} (red) signals with a parallel capacitor clearly displays an RC delay at 1 kHz. Flat DC rectification appears at higher than 10 kHz. Flat DC V_{out} level decreases from 0.6 V to 0.25 due to the coaxial cable-induced ωL_{ext} effect as the frequency increases.

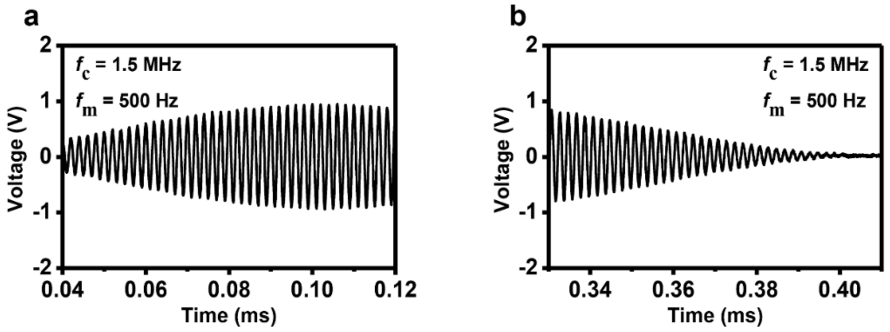

Supplementary Figure 8 Amplitude modulation. **a, b** AM input ascending (**a**) and descending (**b**) voltage band spectra in time domain, generated by function generator at a carrier frequency (f_c = 1.5 MHz) mixed with a modulating frequency (f_m = 500 Hz). AM modulation in the frequency mixture of 500 Hz and 1.5 MHz could be resolved by reducing the time frame in consideration of the limited equipment capacity.

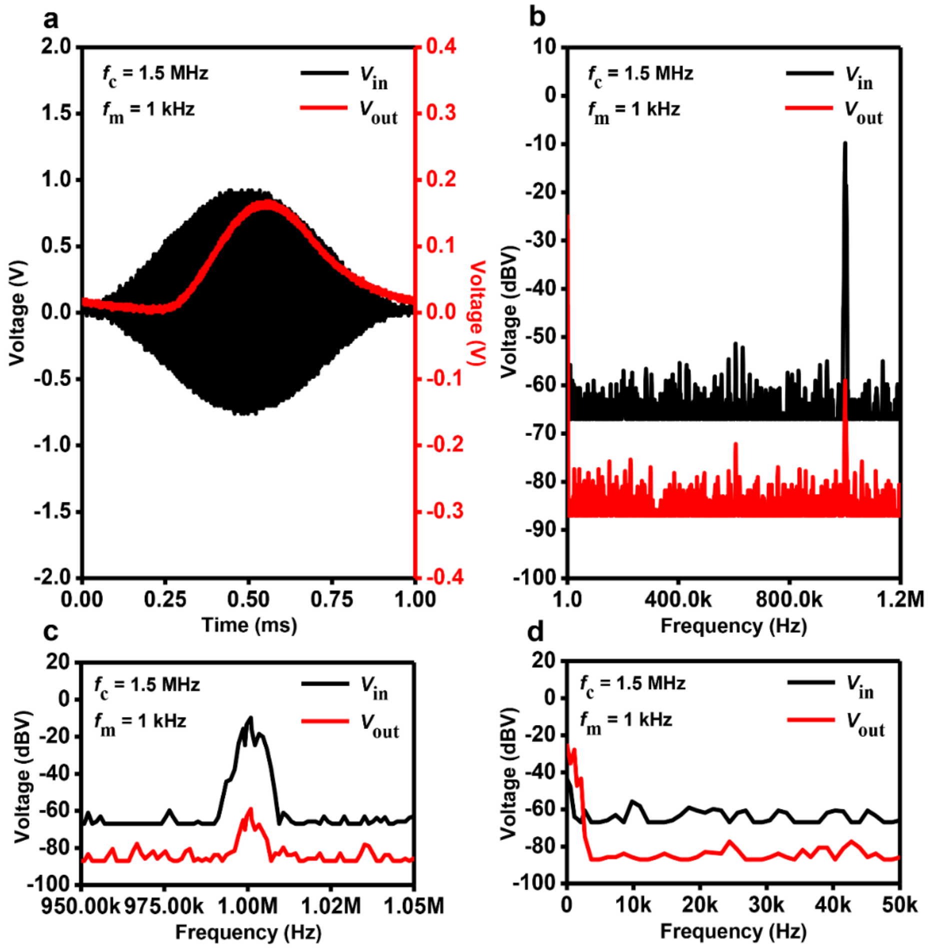

Supplementary Figure 9 AM radio receiver. **a** Amplitude modulated input (black) and demodulated output (red) voltage signal spectra in time domain at a carrier frequency (f_c = 1.5 MHz) as mixed with a modulating frequency (f_m = 1 kHz). **b** Fast Fourier transform (FFT) input (black) and output (red) spectra in frequency domain at a carrier frequency (f_c = 1.5 MHz) and a modulating frequency (f_m = 1 kHz). **c** Zoomed FFT spectra for symmetric baseband which shows a central peak at 1.5 MHz and two fine peaks ±1 kHz apart from 1.5 MHz (carrier feed-through signal peak). They also show much reduced AM input which indicates demodulation effects. **d** Zoomed FFT spectra near 1 kHz, showing main peaks of demodulated signal at 0 Hz and 1 kHz along with distortion-induced trivial signal at 2 kHz.

In the main text, Figure 2e and f present the zoomed views of signals near 1.5 MHz and 5 kHz, respectively. First of all, three peaks near 1.5 MHz are seen from both V_{in} and V_{out} plots in Fig. 2e, where in detail two peaks are symmetrically ±5 kHz apart from central peak at 1.5 MHz. However, in dB (voltage) level, V_{out} appears much diminished compared with V_{in}. As expected, Figure 2f shows the low frequency demodulation signals only from the V_{out} plot, where the main signals at 0 Hz and 5 kHz are observed but along with trivial harmonic distortion-induced signals at 10 kHz and 15 kHz. Such trivial harmonic distortion seems attenuated with the low information frequencies that were used (among 0.5 kHz, 1 kHz, and 5 kHz). We could thus observe more improved demodulation in the case of (1 kHz, 1.5 MHz) combination in Supplementary Fig. 9.

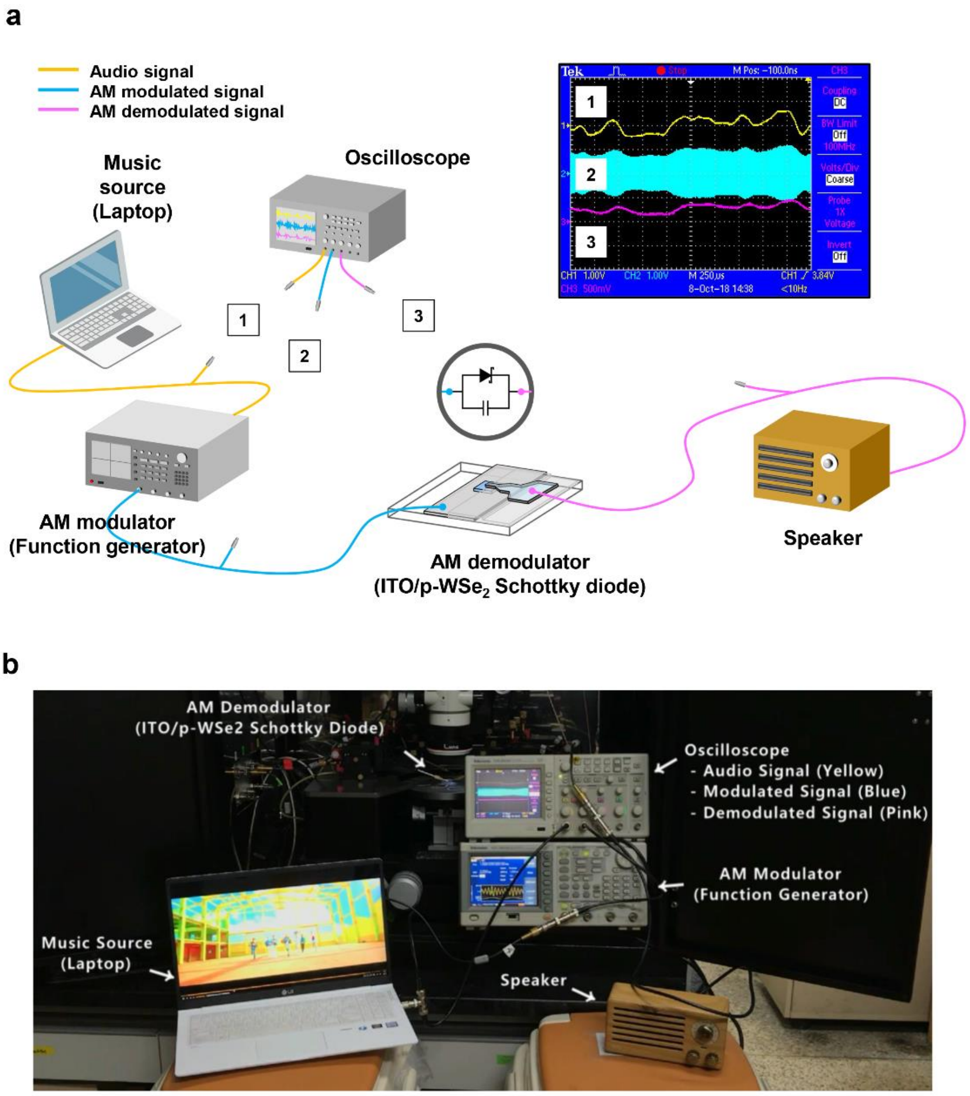

Supplementary Figure 10 Practical AM demodulator. **a** Schematic illustration of practical AM demodulator and oscilloscope-captured display (inset) of audio (yellow), function generated amplitude modulation (skyblue), and demodulation (pink) signals. **b** Photograph of AM demodulator measurement setup. The audio/music is initially played without sounds as directly connected to the oscilloscope, and then it comes out as sounds almost perfect through AM demodulator and speaker.

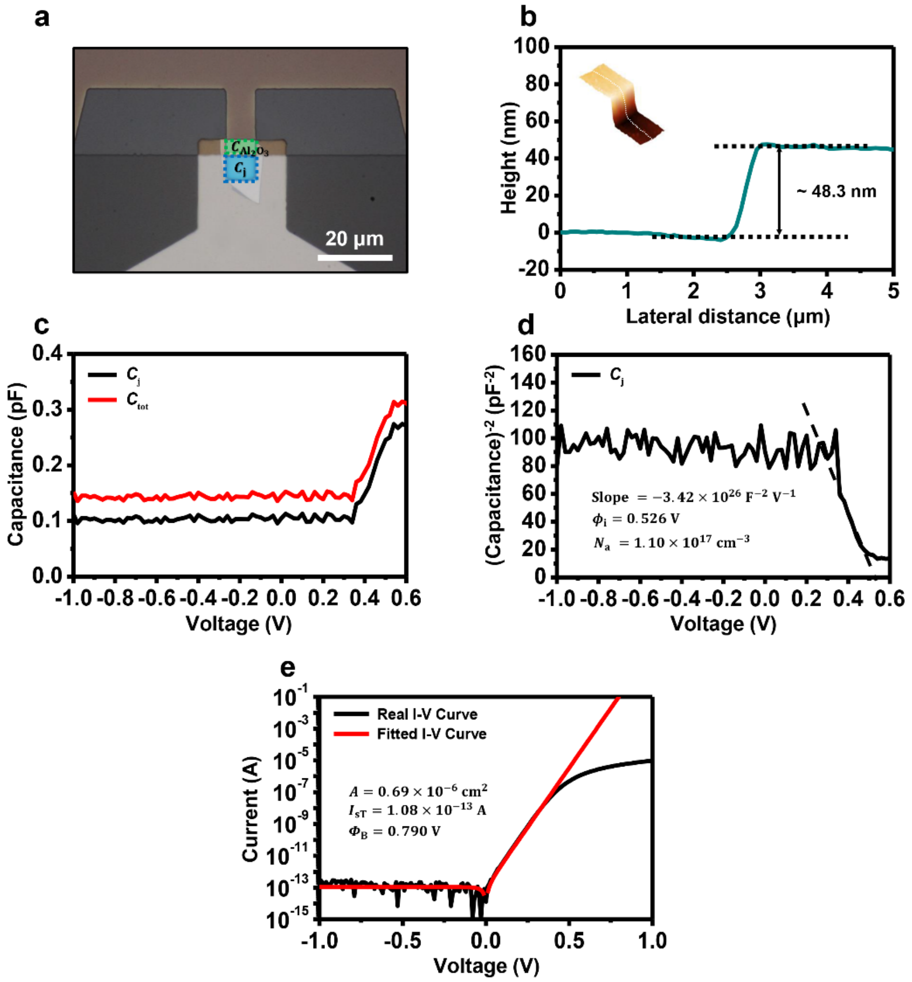

Supplementary Figure 11 Capacitance-voltage and current-voltage characteristics of Type B Schottky diode with minimum capacitive area for S_{11} parameter measurement. **a** Optical microscopic (OM) image showing the separation of junction capacitance area (blue) and oxide capacitance area (green). **b** Atomic force microscopy (AFM) scan image/thickness profiles are shown for the WSe_2 flake in Fig. 3c. **c** Capacitance-voltage (*C*-*V*) characteristics of ITO/p-WSe_2 Schottky diode with (dashed) and without (solid) parallel Al_2O_3–induced capacitance (C_{ox} is less than 40 fF). **d** $1/C^2$-V characteristics of ITO/p-WSe_2 Schottky diode without (solid) parallel Al_2O_3–induced capacitance show approximate hole concentration N_a of 1.1x10^{17} cm^{-3} along with an approximate built-in potential Φ_i of 0.53 V. **e** Current-voltage (*I*-*V*)

characteristics. The *I-V* characteristics of the Schottky diodes can be modeled by the ideal diode equation, $I = I_{sT}[\exp\{q(V-IR_s)/(\eta k_B T)\}-1]$, where $I_{sT} = AA^* T^2 \exp(-q\Phi_B/k_B T)$. A^* (= ~27.6 A cm^{-2} K^{-2}) is the effective Richardson constant, A is the junction area of the diode, η is the ideality factor, Φ_B is the Schottky barrier, and R_s is the series resistance composed of semiconductor resistance (R_j) and contact resistance (R_c). According to detailed analysis in Supplementary Figs. 11d and e, hole concentration p in WSe_2 is 1.1x10^{17} cm^{-3}, built-in potential Φ_i is 0.53 V, and Φ_B is 0.79 V as approximated from 48.3 nm-thick WSe_2 Schottky diode (these numbers are consistent to those of the Type A diode in Fig. 1).

$$I = I_{sT}\left[\exp\left\{\left(\frac{q}{\eta k_B T}\right)(V - IR_s)\right\} - 1\right] \quad \textit{where} \quad I_{sT} = AA^* T^2 \exp\left(-\frac{q\Phi_B}{k_B T}\right)$$

$$\eta = \left(\frac{q}{k_B T}\right)\left[\frac{dV}{d(\ln I)}\right] = \frac{1}{(8.617343 \times \ \ \mathrm{K}^{-1})(300\ \mathrm{K})(34.63826\ \mathrm{V}^{-1})} = 1.12$$

$$A^* = \frac{4\pi q k_B^2 m^*}{h^3} = \frac{4\pi q k_B^2 (0.46 m_e)}{h^3} = 27.6\ \mathrm{A\ cm^{-2}\ K^{-2}}$$

$$\Phi_B = \frac{k_B T}{q}\ln\left(\frac{AA^* T^2}{I_{sT}}\right) = (0.026\ \mathrm{V}) \cdot \ln\left[\frac{(0.69 \times 10^{-6}\ \mathrm{cm^2})(27.6\ \mathrm{A\ cm^{-2}\ K^{-2}})(300\ \mathrm{K})^2}{(1.08 \times 10^{-13}\ \mathrm{A})}\right]$$

$$= 0.79\ \mathrm{V}$$

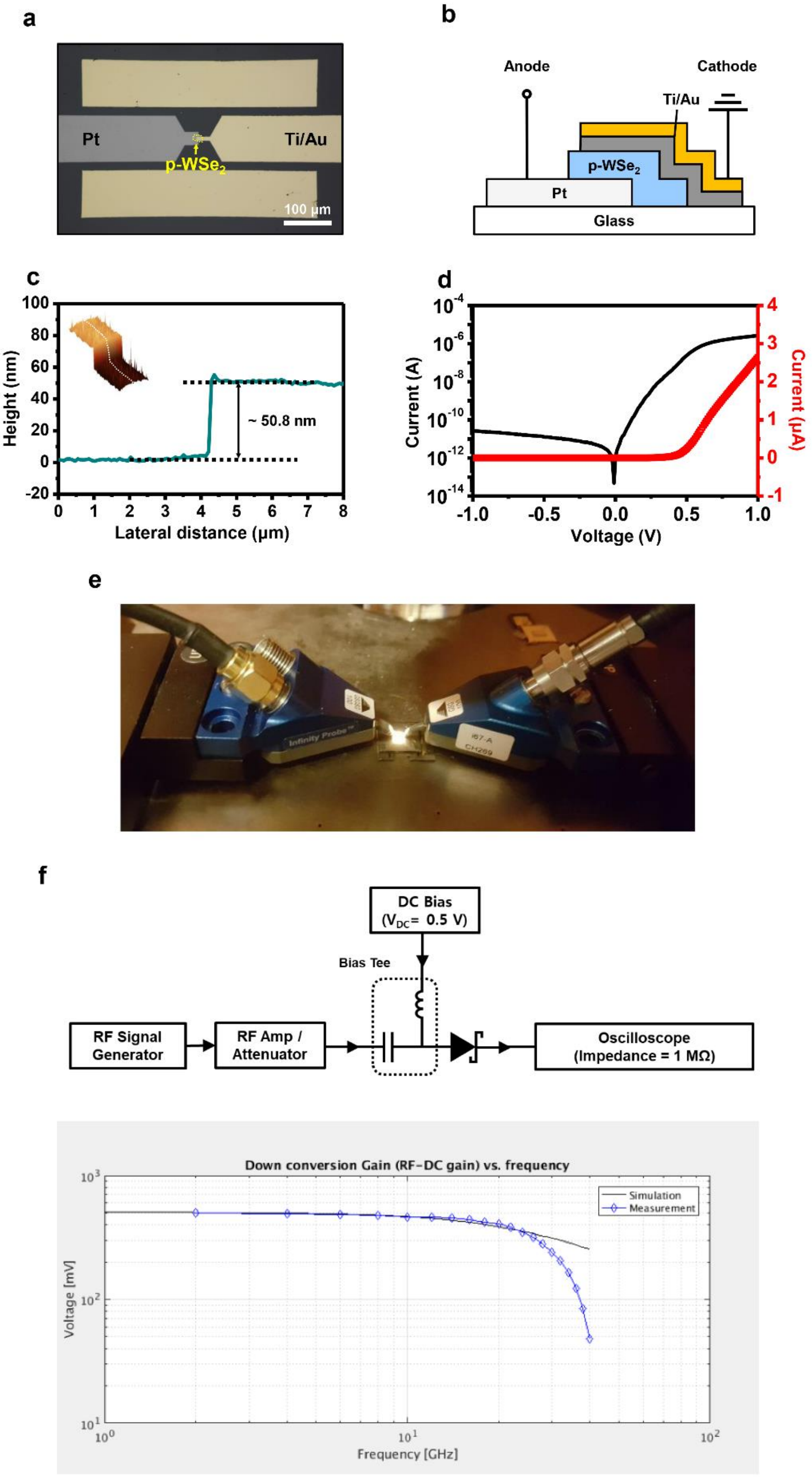
a
Pt
Ti/Au
p-WSe$_2$
100 µm
b
Anode
Cathode
Ti/Au
p-WSe$_2$
Pt
Glass
c
Height (nm)
~ 50.8 nm
Lateral distance (µm)
d
Current (A)
Current (µA)
Voltage (V)
e
f
DC Bias
(V_{DC}= 0.5 V)
Bias Tee
RF Signal Generator
RF Amp / Attenuator
Oscilloscope (Impedance = 1 MΩ)
Down conversion Gain (RF-DC gain) vs. frequency
Simulation
Measurement
Voltage [mV]
Frequency [GHz]

Supplementary Figure 12 DC voltage output measurement to characterize the rectification performance of the two-port architecture WSe_2 Schottky diode. **a, b** Optical microscopic image (**a**) and schematic diagram (**b**) of the two-port architecture Ti/p-WSe_2 Schottky diode prepared on the CPW configuration. **c** Atomic force microscopy (AFM) scan image/thickness profiles are shown for the WSe_2 flake in Supplementary Fig. 12a. **d** Current-voltage (*I*-*V*) characteristics of the WSe_2 Schottky diode. **e** Photographic image of two terminal experiments using two RF probes. **f** Input RF power was given as 10 dBm (10 mW), and RF input voltage amplitude, V_{amp} = 1V ($V_{p\text{-}p}$ = 2V). As shown above scheme of the circuit, terminal 1 was connected to RF output of the bias tee (V_{DC} = 0.5 V) while terminal 2 was connected to an oscilloscope with 1MΩ self-impedance. Then, rectified DC voltage was measured by sweeping RF frequencies. Initial rectified output voltage appears to be 500 mV and cutoff frequency is located at ~25 GHz as 350 mV (~70 % of 500 mV). We conducted these experiments with Ti Schottky contact and without isolation oxide, only to confirm the cutoff frequency at a similar WSe_2 thickness.

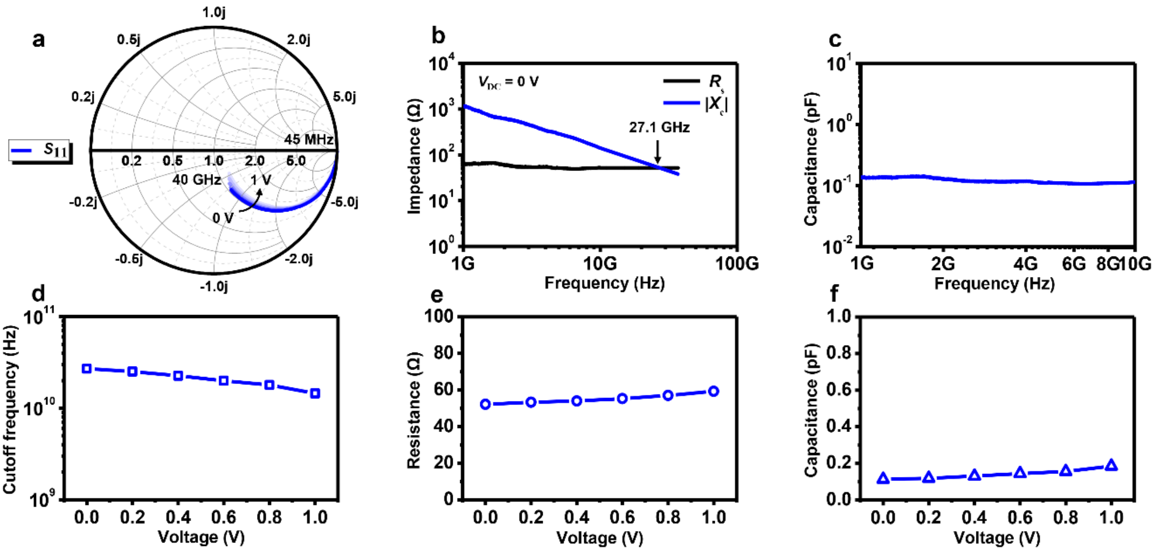

Supplementary Figure 13 S_{11} parameter measurement and resistive/capacitive components of ITO/p-WSe_2 Schottky diode with 48.3 nm-thick WSe_2 flake. **a** S_{11} parameters from 45 MHz to 40 GHz at different bias voltages. As a positive voltage is applied, the blue curve goes inward. **b** Resistance (R_s) and capacitive reactance ($|X_c| = 1/\omega C_{tot}$) plots as a function of frequency under zero external bias (battery-free). Plots are without de-embedding. **c** Capacitance values extracted from $|X_c|$ (= $1/\omega C_{tot}$) plots. **d** Cutoff frequency (f_{cutoff}) measured under different biasing conditions from 0 V to +1 V. With the bias increases, cutoff frequency gradually decreases from 27 to 14 GHz, owing to depletion thickness decrease and C_j increase. **e** Series resistance (R_s) obtained from S_{11} parameter measurements at different biases from 0 V to +1 V. Almost no difference of R_s is seen between 0 V and +1 V. **f** Total capacitance slightly increases with the bias to the ITO/p-WSe_2 Schottky diode as obtained from S_{11} parameter measurement.

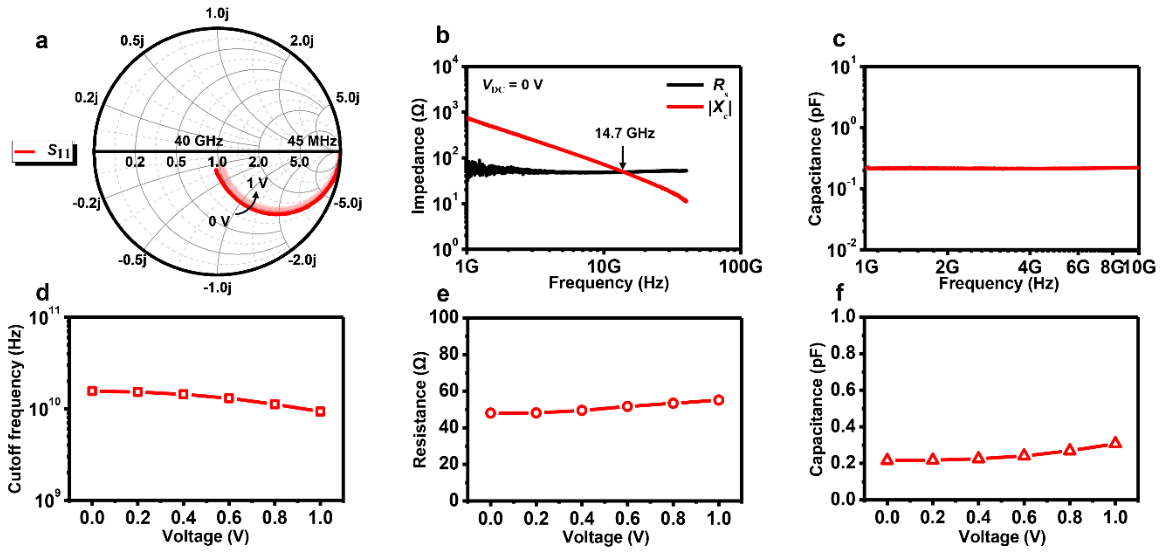

Supplementary Figure 14 S_{11} parameter measurement and resistive/capacitive components of ITO/p-WSe_2 Schottky diode with 23.6 nm-thick WSe_2 flake. **a** S_{11} parameters from 45 MHz to 40 GHz at different bias voltages. As a positive voltage is applied, the red curve goes inward. **b** Resistance (R_s) and capacitive reactance ($|X_c| = 1/\omega C_{tot}$) plots as a function of frequency under zero external bias (battery-free). Plots are without de-embedding. **c** Capacitance values extracted from $|X_c|$ ($= 1/\omega C_{tot}$) plots. **d** Cutoff frequency (f_c) measured under different biasing conditions from 0 V to +1 V. **e** Series resistance (R_s) obtained from S_{11} parameter measurements at different biases from 0 V to +1 V. **f** Total capacitance-voltage characteristics of the ITO/p-WSe_2 Schottky diode obtained from S_{11} parameter measurement. Cutoff frequency decreases from 15 to 10 GHz under forward bias, because of depletion thickness decrease (C_j increase).

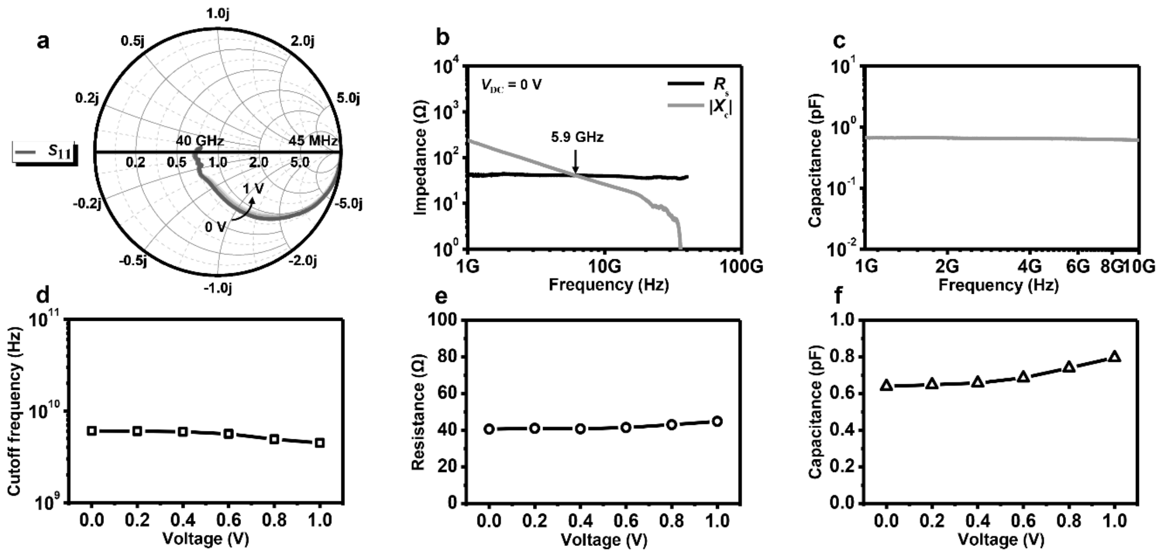

Supplementary Figure 15 S_{11} parameter measurement and resistive/capacitive components of ITO/p-WSe_2 Schottky diode with 7.53 nm-thick WSe_2 flake. **a** S_{11} parameters from 45 MHz to 40 GHz at different bias voltages. As a positive voltage is applied, the black curve goes inward. **b** Resistance (R_s) and capacitive reactance ($|X_c| = 1/\omega C_{tot}$) plots as a function of frequency under zero external bias (battery-free). Plots are without de-embedding. **c** Capacitance values extracted from $|X_c|$ (= $1/\omega C_{tot}$) plots. **d** Cutoff frequency (f_c) measured under different biasing conditions from 0 V to +1 V. **e** Series resistance (R_s) obtained from S_{11} parameter measurements at different biases from 0 V to +1 V. **f** Total capacitance-voltage characteristics of the ITO/p-WSe_2 Schottky diode obtained from S_{11} parameter measurement. Cutoff frequency decreases from 6 to 4 GHz under forward bias, because of depletion thickness decrease (C_j increase).

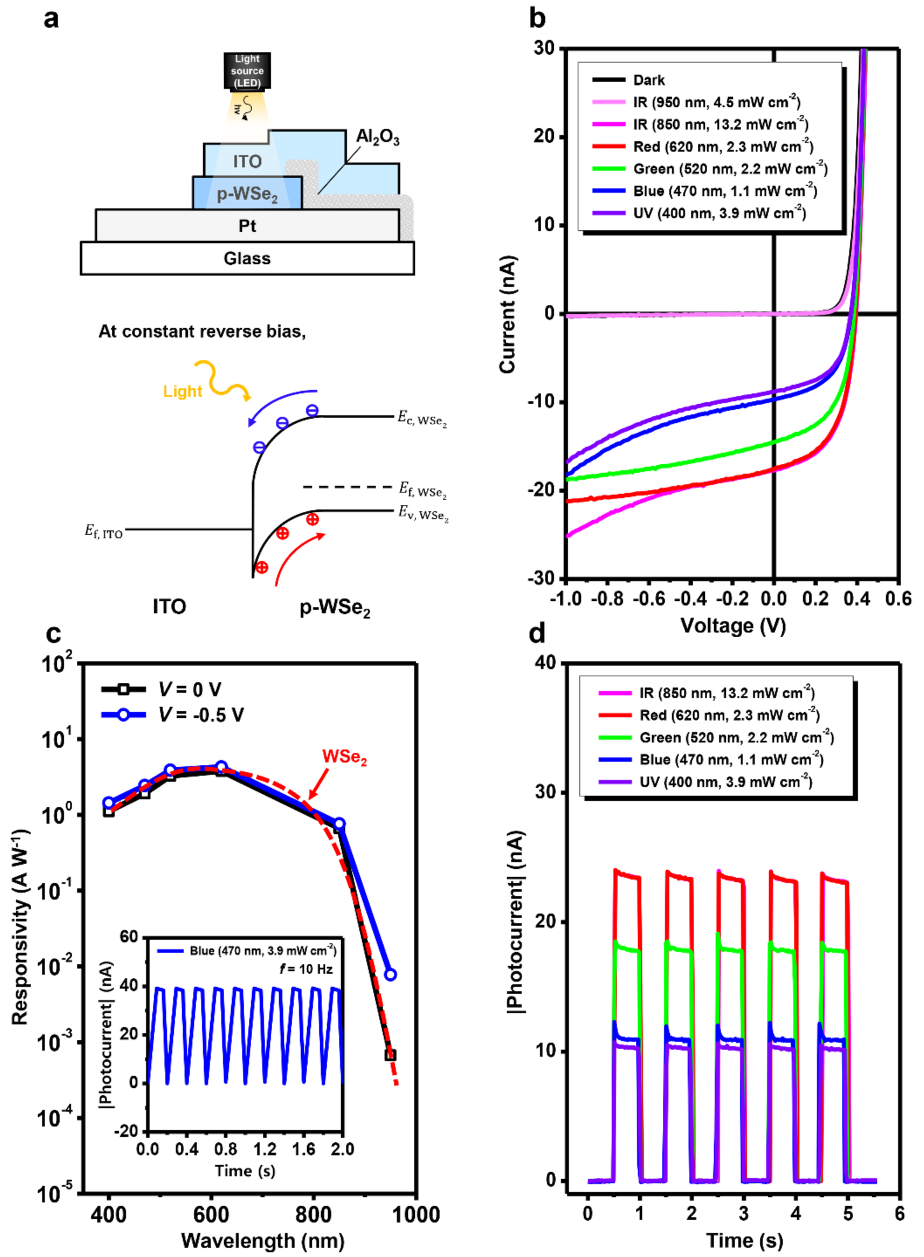

Supplementary Figure 16 Photoelectric and photovoltaic characteristics of ITO/p-WSe_2 Schottky photodiode. **a** Schematic device cross section and band diagram of ITO/p-WSe_2 Schottky photodiode at constant reverse bias. **b** Photoinduced current-voltage (I_{ph}-V) characteristics of our photodiode under NIR (850 nm), red (620 nm), green (520 nm), blue (470 nm), and UV (400 nm) LED illumination. **c** Responsivity plot at a zero bias and a reverse bias

of -0.5 V and temporal photocurrent characteristics (inset) under periodic blue (470 nm, 3.9 mW cm^{-2}) illumination at 10 Hz. The photoelectric and photovoltaic properties are well noted in the visible range, but the near-infrared (NIR) of 950 nm wavelength is seldom detected showing very small responsivity value (less than ~ 10^{-3} A W^{-1}). This means that our WSe_2 semiconductor has its own band gap larger than the photon energy of 950 nm (~ 1.3 eV). **d** Temporal (1 Hz) photocurrent characteristics of the photodiode at a constant reverse bias of -0.5 V under LED illumination. The diode shows quite fast photodynamics with a few tens ms (< 50 ms) of response time.

Supplementary Tables

Supplementary Table 1 Type and device performance comparison of high frequency rectifying vertical diodes.

Material		Device Type	Substrate	Num. of Layer	*t* (nm)	V_T (V)	η	J_{on} (A cm^{-2})	On/Off Ratio	μ (cm^2 V^{-1} s^{-1})	f_{cutoff} (GHz)	C_j (pF)	R_{ct} (Ω μm^2)	Ref.
Nanomaterial	MoS_2*	Schottky diode	Sapphire	FL⁺	1-3	0.35	1.39	0.10 (A cm^{-1})	10^3	10-100	4.0	0.038	-	[1]
		Schottky diode	PET	FL⁺	1-3	0.32	1.50	0.03 (A cm^{-1})	10^2	10-100	10	0.004	56 (kΩ μm)	[2]
	WSe_2	PIN diode	Si/SiN	ML⁺	12	0.40	1.45	3.1×10^5	3.0×10^2	-	0.30	2.2	-	[3]
		Schottky diode	Glass	ML⁺	50	0.50	1.12	14	10^8	2.2	27	0.10	3400	Our work
Metal oxide semiconductor	IGZO	Schottky diode	Glass	Thin-film	50	0.56	1.52	20	10^4	5-10	8.0	1.0	5200	[4], [5]
			PET	Thin-film	80	0.43	2.10	400	10^3	5-10	5.9	0.81	1.3×10^4	
Organic semiconductor	Pentacene	Schottky diode	Glass	Thin-film	120	0.74	1.51	0.80	10^7	0.11	1.2	1.7	4.9×10^5	[6]
	C_{60}	Schottky diode	Glass	Thin-film	100	0.69	1.36	47	4.6×10^4	0.42	0.70	14	9.2×10^5	[7]
Conventional semiconductor	sc-Si	Schottky diode	Si	Bulk	Wafer (>10 μm)	0.30	1.80	1.0×10^5	10^3	1400	400	0.043	1.8	[8], [9]
	sc-Si:μPs	Schottky diode	PET	Thin-film	4000	1.00	2.90	20	10^2	1400	1.6	-	-	[10]
	nc-Si:H	Schottky diode	Glass	Thin-film	750	0.73	1.70	10	10^3	5.4	0.11	7.0	2.0×10^6	[11], [12]
	a-Si:H	Schottky diode	Glass	Thin-film	1000	0.79	1.20	1.0×10^{-3}	10^5	1.0	0.005	80	4.0×10^8	[13], [14]
	GaAs	Schottky diode	GaAs	Bulk	Wafer (>10 μm)	<0.60	1.70	1.5×10^6	2.0×10^2	8800	3200	-	3.5	[8], [15], [16]

*The MoS_2-based diode is a lateral in-plane Schottky diode with a 1T-2H metallic-semiconducting phase junction.

⁺Each abbreviation stands for few- and multi-layers.

Supplementary References

1. Zhang, X. et al. MoS_2 Phase-junction-based Schottky Diodes for RF Electronics. *IEEE/MTT-S International Microwave Symposium* **1**, 345–347 (2018).
2. Zhang, X. et al. Two-dimensional MoS_2-enabled flexible rectenna for Wi-Fi-band wireless energy harvesting. *Nature* **566**, 368–372 (2019).
3. Nazir, G. et al. Ultimate limit in size and performance of WSe_2 vertical diodes. *Nat. Commun.* **9**, 5371, https://doi.org/10.1038/s41467-018-07820-8 (2018).
4. Min, S. W., Yoon, M., Yang, S. J., Ko, K. R. & Im, S. Charge-Transfer-Induced p-Type Channel in MoS_2 Flake Field Effect Transistors. *ACS Appl. Mater. Interfaces* **10**, 4206–4212 (2018).
5. Zhang, J. et al. Flexible indium–gallium–zinc–oxide Schottky diode operating beyond 2.45 GHz. *Nat. Commun.* **6,** 7561, https://doi.org/10.1038/ncomms8561 (2015).
6. Kang, C. et al. 1 GHz Pentacene Diode Rectifiers Enabled by Controlled Film Deposition on SAM-Treated Au Anodes. *Adv. Electron. Mater.* **2**, 1500282 (2016).
7. Im, D., Moon, H., Shin, M., Kim, J. & Yoo, S. Towards Gigahertz Operation: Ultrafast Low Turn-on Organic Diodes and Rectifiers Based on C_{60} and Tungsten Oxide. *Adv. Mater.* **23**, 644–648 (2011).
8. Muller, R., Kamins, T. & Chan, M. *Device Electronics for Integrated Circuits* (John Wiley & Sons Inc., 2003).
9. Sankaran, S., K. O., K. Schottky diode with cutoff frequency of 400 GHz fabricated in 0.18 μm CMOS. *Electron. Lett.* **41**, 8 (2005).
10. Sani, N. et al. All-printed diode operating at 1.6 GHz. *Proc. Natl. Acad. Sci. U. S. A.* **111**, 11943–11948 (2014).
11. Stieler, D., Dalal, V. L., Muthukrishnan, K., Noack, M. & Schares, E. Electron mobility in nanocrystalline silicon devices. *J. Appl. Phys.* **100**, 036106 (2006).

12. Sanz-Robinson, J. et al. Hybrid Amorphous/Nanocrystalline Silicon Schottky Diodes for High Frequency Rectification. *IEEE Electron Device Lett.* **35**, 425–427 (2014).

13. Sanz-Robinson, J., Rieutort-Louis, W., Verma, N., Wagner, S. & Sturm, J. C. Frequency Dependence of Amorphous Silicon Schottky Diodes for Large-area Rectification Applications. *Device Res. Conf. - Conf. Dig. DRC* **200**, 135–136 (2012).

14. Hull, R., Osgood, R. M., Sakaki, H., & Zunger, A. *Technology and applications of Amorphous Silicon*. (Springer, 2003).

15. Bulcha, B. T. et al. Design and Characterization of 1.8-3.2 THz Schottky-Based Harmonic Mixers. *IEEE Trans. Terahertz Sci. Technol.* **6**, 737–746 (2016).

16. McEvoy, A., Markvart, T. & Castaner, L. *Practical Handbook of Photovoltaics - Fundamentals and Applications* (Academic Press, 2012).
